# Response to ‘Increase of nerve growth factor levels in the human herniated intervertebral disc: can annular rupture trigger discogenic back pain?’ – authors’ reply

**DOI:** 10.1186/s13075-015-0608-3

**Published:** 2015-04-08

**Authors:** Yasuchika Aoki, Arata Nakajima, Seiji Ohtori, Hiroshi Takahashi, Fusako Watanabe, Masato Sonobe, Fumiaki Terajima, Masahiko Saito, Kazuhisa Takahashi, Tomoaki Toyone, Atsuya Watanabe, Takayuki Nakajima, Makoto Takazawa, Koichi Nakagawa

**Affiliations:** Department of Orthopaedic Surgery, Toho University Sakura Medical Center, 564-1 Shimoshizu, Sakura, Chiba 285-8741 Japan; Department of Orthopaedic Surgery, Eastern Chiba Medical Center, 3-6-2 Okayamadai, Togane, Chiba 283-8686 Japan; Department of Orthopaedic Surgery, Graduate School of Medicine, Chiba University, 1-8-1 Inohana, Chuo-ku, Chiba-city, Chiba 260-8677 Japan; Department of Orthopaedic Surgery, Teikyo University Mizonokuchi Hospital, 3-8-3 Mizonokuchi, Takatsu-ku, Kawasaki, 213-8507 Japan

We appreciate the interest and attention paid by Agilli and Ekinci, and we agree with their point that nerve growth factor (NGF) levels could be affected by multiple factors [1]. As pointed out, the paper would be improved by considering the effects of these factors; thus the data were re-examined. This analysis found that the number of patients who had neuropsychiatric diseases and use medications (one patient in the herniated group and two patients in the nonherniated group) and had diabetes mellitus (two patients in each group) were similar between the two groups. As Bullo and colleagues described, obesity is one of the important factors affecting NGF levels in blood samples [2]. These authors also indicated the influence of body mass index (BMI) on NGF levels; thus, the BMI of our patients was analyzed and included in the multivariate analysis. The BMI of the two groups showed no significant difference (24.0 ± 4.0 in the herniated group and 25.3 ± 3.2 in the nonherniated group, *P* = 0.20), and Pearson’s correlation analysis showed no significant correlation between the level of NGF and BMI (*P* = 0.91). The multivariate analysis adjusted for age, sex, disc degeneration and BMI still showed a significant correlation between disc herniation and NGF levels (*P* = 0.018).

A previous report of a rat study described that disc injury induced an inflammatory response and NGF upregulation in the disc [3]. It has also been reported that inflammatory mediators including NGF were increased in human symptomatic discs [4,5]. The intervertebral disc is avascular, and metabolic transport depends on diffusion through the vertebral endplates [6]. This limited molecular transport may explain why NGF did not increase in discs from our obese patients. These observations suggest that the significant increase of NGF in herniated discs was due to local tissue reaction following annular rupture. Because there may be many factors affecting NGF levels in the disc, it would be difficult to include them all in a multivariate analysis. However, as Agilli and Ekinci indicated, several important factors should be taken into account when studying NGF levels in the disc.

## Abbreviations

BMI: Body mass index
NGF: Nerve growth factor
